# Benefits of neutral polysaccharide from rhizomes of *Polygonatum sibiricum* to intestinal function of aged mice

**DOI:** 10.3389/fnut.2022.992102

**Published:** 2022-09-20

**Authors:** Li-Xia Li, Xin Feng, Meng-Ting Tao, Berit Smestad Paulsen, Chao Huang, Bin Feng, Wei Liu, Zhong-Qiong Yin, Xu Song, Xinghong Zhao, Xiao-Xia Liang, Li-Zi Yin, Hua-Qiao Tang, Yuan-Feng Zou

**Affiliations:** ^1^Natural Medicine Research Center, College of Veterinary Medicine, Sichuan Agricultural University, Chengdu, China; ^2^Department of Pharmacy, Section Pharmaceutical Chemistry, Area Pharmacognosy, University of Oslo, Oslo, Norway; ^3^Laboratory of Experimental Animal Disease Model, College of Veterinary Medicine, Sichuan Agricultural University, Chengdu, China; ^4^Animal Nutrition Institute, Sichuan Agricultural University, Chengdu, China; ^5^Key Laboratory of the Ministry of Education for the Standardization of Traditional Chinese Medicine, Pharmacy College, Chengdu University of Traditional Chinese Medicine, Chengdu, China; ^6^Key Laboratory of Animal Disease and Human Health of Sichuan Province, College of Veterinary Medicine, Sichuan Agricultural University, Chengdu, China

**Keywords:** heteropolysaccharide, *Polygonatum sibiricum*, anti-aging, jejunum, colon

## Abstract

One purified neutral polysaccharide fraction was obtained from the rhizome of *Polygonatum sibiricum* by DEAE ion exchange and gel chromatography. Structure elucidation was performed by methanolysis, methylation, FT-IR, and NMR. The results indicated that PSP-NP was composed of 1,4-β-D-Gal,1, 4, 6-β-D-Gal, T-α-D-Man,1, 4-α-D-Glc, and T-α-D-Glc with a molecular weight of 43.0 kDa. We supplied this polysaccharide to aged mice and found it is of benefits to intestinal functions, as indicated by better tissue integrity and motility, improved oxidative stress and inflammation, reduced intestinal permeability and serum LPS level, as well as balanced gut microbial composition and short-chain fatty acids production. These results display a novel *Polygonatum sibiricum* polysaccharide to improve the intestinal function of aged mice, which provides pieces of evidence for its further development and utilization.

## Introduction

Aging is a process that accumulates detrimental changes to cellular components and thus affects tissue homeostasis, resulting in disease or increased risk of morbidity and mortality (1). Previously work revealed tightly integration between aging and chronic inflammation, and multiple aging-related factors are implicated, such as redox imbalance, mitochondrial damages, senescence of the immune/endocrine system (2), etc. The gastrointestinal tract is an important organ of body immunity, the mucosal immune system of which provides the first defense-line against environmental insults and pathogens (3). The gastrointestinal tract, especially the intestine, couples signals from gut microbiota, metabolites, stress, and immune response and is implicated in controlling immune defense not just itself but also elsewhere in the body (4, 5). As aging compromises the intestinal immune response that affects innate and adaptive immunity and subsequently leads to detrimental systemic effects on the entire organism, improvements to intestinal function could bring about benefits to aging-related defects.

*Polygonati Rhizoma*, the rhizome from the Liliaceae Polygonatum plant that is widely distributed throughout China with more than 30 species (2, 6). *Polygonati Rhizoma* has been used as food and medicine in China for more than 2000 years, and the beneficial effects of which are very accurate in Chinese clinical practice (7, 8). Three of *Polygonati Rhizoma* are now listed in Chinese Pharmacopeia as Traditional Chinese Medicine, including the rhizome of *Polygonatum sibiricum*, *P. kingianum* Coll. et Hemsl and *P. cyrtonema* Hua. *Polygonati Rhizoma* has a wide range of pharmacological effects of anti-diabetes, anti-fatigue, Antioxidant, antimicrobial as well as anti-aging and immune improvement (9–11). While a variety of bioactive compositions like steroidal saponins, triterpenes, flavones, phytosterols and volatile oils have been isolated from *Polygonati Rhizoma*, polysaccharides are thought to be one of the most important bioactive-compounds from it (12). Multiple polysaccharides have been isolated from these three species of *Polygonati Rhizoma* with different methods, but most of them lack systematic research on isolation, purification and their bio-activity, and the comparative studies on neutral polysaccharide components in different species have also not been reported (7, 13). Most importantly, the function of *Polygonati Rhizoma* polysaccharides in the intestine, where polysaccharides are degraded and display their bioactivities, is not well understood.

Here in this study, we aim to isolate and purify the polysaccharides from the rhizome of *Polygonatum sibiricum*, *P. kingianum* Coll. et Hemsl and *P. cyrtonema* Hua. *Polygonati Rhizoma* by DEAE ion exchange chromatography and gel filtration, screen polysaccharides with high bioactivities *in vitro*, and then structurally characterize them and study their benefits to the intestine of aged mice.

## Materials and methods

### Materials

*Polygonatum sibiricum* was collected from Baiyun Mountain, Luoyang City, Henan Province, China. *P. kingianum* was collected from Mile Mountain, Mile City, Yunnan Province, China. *P. cyrtonema* was collected from Longcanggou, Yingjing County, Yaan City, Sichuan Province, China. It was identified as *Polygonatum sibiricum* by Dr. Lixia Li, College of Veterinary Medicine, Sichuan Agricultural University. The rhizomes of all materials were washed, sliced, steamed for 60 mins, and then dried at 50°C.

Standard acetic acid, propionic acid, and butyric acid were purchased from Sigma-Aldrich. EMB medium, MRS medium, and BS medium were obtained from Hope Bio Biotechnology Co., Ltd. (Qingdao, China). H&E and AB-PAS stain kits were purchased from Solarbio (Beijing, China). RevertAid First Strand cDNA was purchased from Thermo Fisher Scientific. SYBR Green Supermix was purchased from Bio-Rad.

The standards of D-Mannose (Man); L-Rhamnose (Rha); D-Glucuronic acid (GlcA); D-Galactose (Gal); D-Glucose (Glc); D-Galacturonic acid (GalA); D-Fucose (Fuc); D-Xylose (Xyl); L-Arabinose (Ara); and D-Fructose (Fru) were purchased from Solarbio (Beijing, China). All other chemicals, such as phenol, sulfuric acid, trichloromethane, isopropyl alcohol, ethanal, etc., were all of analytical grade and obtained from the Kelong Chemical factory (Chengdu, China).

### Extraction of polysaccharides from *Polygonatum sibiricum*

Processed rhizomes from *P. sibiricum*, *P. kingianum*, and *P. cyrtonema* were extracted by 95% and 50% ethanol separately to remove low-molecular-weight and different-polarity lipophilic compounds. Then dried residuals were extracted twice with boiling water to obtain crude extracts. Those extracts were concentrated (RE-3000, Yarong Bio-chemical Instruments Factory, Shanghai, China) and precipitated by absolute ethanol. After lyophilized, the crude polysaccharide was obtained and named PSP, PKP, and PCP.

### Purification of polysaccharides from *Polygonatum sibiricum*

The crude polysaccharide (400 mg) was dissolved in distilled water (20 mL), and applied to a DEAE Sepharose (Fast Flow, FF) column (50 mm × 40 cm, Beijing Rui Da Heng Hui Science Technology Development Co., Ltd., Beijing, China) after having been filtered through a 0.45 μm filter. The neutral fraction was eluted with a 3-fold column volume (approximately 2 L) of distilled water at a speed of 2 mL/min. Afterward, the acidic fraction was further eluted with linear gradient NaCl solution (0∼1.5 M) with a flow rate 2 mL/min. The related fractions were pooled together based on the phenol-sulfuric acid assay, dialyzed at a cut-off of 3500 Da against distilled water to remove NaCl, and lyophilized. Then, it was dissolved in 10 mM NaCl buffer and purified by gel filtration on a Sepharose 6FF column (2.5 cm × 100 cm, Beijing Rui Da Heng Hui Science Technology Development Co., Ltd., Beijing, China) after filtration through a 0.45 μm filter, and eluted with 10 mM NaCl at 1.0 mL/min. Both neutral and acid fractions were pooled based on the phenol-sulfuric acid assay, dialyzed, and lyophilized, and the neutral fractions were named PSP-NP, PKP-NP, and PCP-NP, while the acidic fractions were named PSP-AP, PKP-AP, and PCP-AP.

### Cell culture, cell viability assay, and quantitative real-time PCR

The porcine epithelial (IPEC-J2) cells were cultured in DMEM (Gibco) medium containing 10% FBS (Gibco) in an incubator with the atmosphere of 5% CO_2_ at 37°C. Cells were plated in 6-well cell plates (1 × 10^6^ cells per well), and the CCK-8 solution (10 μL per well) was added co-culturing for 1 h after adding polysaccharide (at the final concentration of 5 and 10 μg/mL) at the plates for 12 h. The absorbance of each well was finally read at 450 nm using a microplate reader for the cell viability assay. In the meantime, after the polysaccharide was supplied, the cells were collected to extract the total RNA by lysing with Trizol Regent according to the manufacturer’s instructions. The concentration was measured with a spectrophotometer (NanoDrop 2000, Thermo Fisher Scientific, Shanghai, China) and the quality of RNA was assessed by agarose gel. Then the cDNA was obtained by reverse transcription with reverse transcriptase, according to the manufacturer’s instructions (Thermo Fisher Scientific, United States). Quantitative real-time PCR was performed by the Bio-Rad CFX96 system, using GAPDH as an internal reference. Primer sequences are as follow: SOD1: Fr 5′-GCAGGGCACCATCTACTTCG-3′, Rv 5′-CTGCACTGGTACAGCCTTGT-3′.

### Characterization of polysaccharide fraction with high antioxidant activity

#### Chemical compositions and linkage determination

According to previous study (14), the methanolysis of the fraction was performed with 3 M hydrochloric acid in anhydrous methanol for 24 h at 80°C, and Mannitol was used as an internal standard. The methylglycosides obtained were converted into their corresponding derivatives − trimethylsilylated (TMS), and then analyzed by gas and then analyzed by gas chromatography (GCThermo Scientific, Milan, Italy). Glycosidic linkage elucidation was performed by methylation studies. Polysaccharide samples were methylated, hydrolyzed, reduced, and acetylated (15). Finally, the derivatives were analyzed by GC-MS using a GCMS-QP2010 (Shimadzu, Kyoto, Japan) attached to a Restek Rxi-5MS column (30 m; 0.25 mm, i.d.; 0.25 μm film). The injector temperature was 280°C; the ion source temperature was 200°C; and the interface temperature was 300°C. The column temperature was 80°C when the sample was injected and then increased 10°C/min to 140°C, followed by 4°C/min to 210°C, and then 20°C/min to 300°C, using helium as the carrier gas (pressure control: 80 kPa). The compound at each peak was characterized by an interpretation of the retention times and the characteristic mass spectra. The relative amount of each type of linkage was determined based on the area of each compound and related to the total amount of each monosaccharide type as determined by methanolysis (16).

#### Molecular weight determination

Determination of homogeneity and molecular weight of polysaccharide was performed by size exclusion chromatography on a HiloadTM 16/60 SuperdexTM 200 prep grade column (GE Healthcare, Uppsala, Sweden) combined with the Äkta system (FPLC, Pharmacia Äkta, Amersham Pharmacia Biotech, Uppsala, Sweden). Dextran polymers (Pharmacia, NJ, United States) of 10, 40, 70, 500, and 2,000 kDa were used as calibration standards (17).

#### FT-IR and NMR spectroscopy

The FT-IR studies were done on a sample after grinding and pressing a mixture of a 1 mg polysaccharides sample with dried KBr into a 1 mm thick disk by a Table Press Machine (Daxiang Electronic Machinery, Guangzhou, China). The spectrum was then recorded in a PerkinElmer FT-IR spectrophotometer (PerkinElmer, Waltham, MA, United States) in the wavelength range of 4000–400 cm^–1^ (18).

The 1H-NMR and 13C-NMR spectra of polysaccharides were recorded in D2O solution on an AV600 spectrometer (600 MHz, Bruker, Rheinstetten, Germany) after a three-time deuterium exchange with D2O by freeze-drying.

### Protective activities of polysaccharide *in vivo*

#### Animal experimental design

All animal studies were performed in accordance with the Guidelines for the Care and Use of Laboratory Animals of Sichuan Province, China (The People’s Government of Sichuan Province, China, 2019) and the Regulations for the Administration of Affairs concerning Experimental Animals (The State Science and Technology Commission of China, Rev 2017-1), and they were reviewed and approved by the Animal Care and Use Committee of Sichuan Agricultural University, with the permit No. DYXY141142115. Sixty-four SPF C57BL/6Cnc mice (10 months old, 28−32 g, male) were purchased from Beijing Vital River Laboratory Animal Technology Co., Ltd. (Beijing, China). The mice were housed in a standard polypropylene cage, given ad libitum access to diet and tap water in a controlled environment with a temperature of 21 ± 1°C, relative humidity of 50–60%, and a 12 h light/12 h dark cycle. After 7 days of acclimatization with free access to food and water, they were randomly divided into 4 groups, (16 mice in each group), one normal control group and three polysaccharide administration groups. The normal control group (Ctr) received intragastric administration with saline. The other three groups received different doses of purified polysaccharides (50, 100, and 200 mg/kg/day), named as low, medium, and high groups, respectively. All mice were successively administered with the above concoctions for 6 weeks. The 24 h after the last drug administration, blood samples were collected. The intestines were rapidly excised and were separated as jejunum and colon. All the samples were dissected into two portions. One portion was frozen in liquid nitrogen and subsequently stored at −80°C for gene expression analysis. And the other one was stored in 4% paraformaldehyde for histopathological evaluation.

#### Histological evaluation and goblet cells analysis

To estimate the pathological changes in the intestinal tissue, H&E staining was conducted for paraffin embedded sections. The jejunum was dehydrated, embedded in paraffin wax, cut into slices with a thickness of 5 μm, and then deparaffinized, rehydrated, and stained with hematoxylin and eosin. The degree of microscopic injury was evaluated on the basis of a previously published scoring system (1; Table 1). The length of the villi and the depth of crypt were then measured by Image-Pro Plus, and ratios were compared. The goblet cells were counted after AB-PAS staining according to the manufacturer’s instructions. After rehydration, tissues were stained with alcian blue, oxidant, Schiff reagent, and hematoxylin. Gene expression of Mucin 2 was measured by qRT-PCR.

**TABLE 1:** Intestinal mucosal injury score.

Grade	Standards
0	Normal mucosal villi
1	Development of subepithelial Gruenhagen’s space at the apex of the villus with capillary congestion
2	Extension of the subepithelial space with moderate lifting of epithelial layer from the lamina propria
3	Massive epithelial lifting down the sides of villi and a few tips may be denuded
4	Dull villi with lamina propria and dilated capillaries exposed and increased inflammatory infiltration of the lamina propria
5	Digestion and disintegration of lamina propria; hemorrhage and ulceration.

#### Determination of the motility function of the intestinal

Total RNA in jejunum and colon was obtained by a Trizol reagent, and cDNA was collected by a reverse transcription with a reverse transcriptase. Quantitative real-time PCR was performed to analyze relative gene expressions of vasoactive intestinal peptide (VIP) in the jejunum and colon using β-actin as an internal reference. Primer sequences are shown in Table 2.

**TABLE 2:** Primer sequences for quantitative real-time PCR.

Prime	5′→3′
β-actin	Fr 5′-GGCTGTATTCCCCTCCATCG-3′
	Rv 5′-CCAGTTGGTAACAATGCCATGT-3′
VIP	Fr 5′-AGTGTGCTGTTCTCTCAGTCG-3′
	Rv 5′ -GCCATTTTCTGCTAAGGGATTCT-3′
Mucin 2	Fr 5′-ATGCCCACCTCCTCAAAGAC-3′
	Rv 5′ -GTAGTTTCCGTTGGAACAGTGAA-3′
ZO-1	Fr 5′- ACCACCAACCCGAGAAGAC-3′
	Rv 5′ - CAGGAGTCATGGACGCACA-3′
Occludin	Fr 5′-TTGAAAGTCCACCTCCTTACAGA-3′
	Rv 5′ -CCGGATAAAAAGAGTACGCTGG-3′
IL-1β	Fr 5′-GAAATGCCACCTTTTGACAGTG -3′
	Rv 5′-TGGATGCTCTCATCAGGACAG-3′
IL-4	Fr 5′- GGTCTCAACCCCCAGCTAGT-3′
	Rv 5′ -GCCGATGATCTCTCTCAAGTGAT-3′
IL-6	Fr 5′-TAGTCCTTCCTACCCCAATTTCC-3′
	Rv 5′ -TTGGTCCTTAGCCACTCCTTC-3′
IL-10	Fr 5′- GCTCTTACTGACTGGCATGAG-3′
	Rv 5′ -CGCAGCTCTAGGAGCATGTG-3′
IL-12A	Fr 5′-CTGTGCCTTGGTAGCATCTATG-3′
	Rv 5′ -GCAGAGTCTCGCCATTATGATTC-3′
IL-12B	Fr 5′-TGGTTTGCCATCGTTTTGCTG-3′
	Rv 5′ -ACAGGTGAGGTTCACTGTTTCT-3′
IL-17	Fr 5′- TTTAACTCCCTTGGCGCAAAA-3′
	Rv 5′ -CTTTCCCTCCGCATTGACAC-3′
IL-23	Fr 5′-ATGCTGGATTGCAGAGCAGTA-3′
	Rv 5′ -ACGGGGCACATTATTTTTAGTCT-3′
TNF-α	Fr 5′-CTGAACTTCGGGGTGATCGG-3′
	Rv 5′-GGCTTGTCACTCGAATTTTGAGA-3′
TGF-β	Fr 5′- CTCCCGTGGCTTCTAGTGC-3′
	Rv 5′- GCCTTAGTTTGGACAGGATCTG-3′

#### Determination of the intestinal mucosal barrier

ELISA assay was conducted to measure the LPS concentration in serum; the gene expressions of mucin; and the tight junction proteins, including ZO-1 and Occludin, were studied by qRT-PCR. Primer sequences are shown in Table 2.

#### Determination of the intestinal immune function

Jejunum and colon were ground by liquid nitrogen in sterile mortars, and then homogenized in 10-fold volumes (g/mL) of PBS. The supernatants were obtained for the sIgA and carbonylated proteins assay using an ELISA kit after centrifuging at 3000 g/min for 10 min.

#### Determination of inflammatory factors in the intestinal

Detected genes of interleukins were *IL-1*β, *IL-2*, *IL-4*, *IL-6*, *IL-10*, *IL-12*, *IL-17*, *IL-23*, *TNF*-α, and *TGF*-β. β-*actin* was used as an internal reference. Primer sequences are shown in Table 2.

#### Influence on gut microbiome

##### Differential cultivation of cecum contents

The fluence of polysaccharides on gut microbiome was evaluated by bacterial counts and short-chain fatty acids (SCFA) in contents of the gut. The contents in cecum were obtained on the last day of this research. They were weighted and homogenized in saline and diluted with 10-fold dilutions from 102 to 107. Each dilutes was added to a culture medium in triplicate for bacterial differential cultivation. Bifidobacterium and Lactobacillus were respectively cultured in MRS and BS agar mediums in an anaerobic incubator with 85% N_2_, 10% H_2_, 5% CO_2_ (Thermo Fisher Scientific 1029, Waltham, MA, United States) at 37°C. *Escherichia coli* were incubated in an EMB agar medium in an aerobic incubator (DHP-9082, Jiecheng Experimental Apparatus Co., Ltd., Shanghai, China). The bacteria count was expressed as log10 CFU (colony-forming unit)/g of cecum content.

##### Determination of short-chain fatty acids

In order to determine the concentration of SCFA in cecum contents, they were weighted and homogenized in 0.15 mL ddH_2_O. They were centrifuged at 10,000 rpm for 10 min to obtain the supernatant, after being placed in room temperature for 30 min. Then, 20 μL 25% metaphosphoric acid and 1.52 μL 210 mmol/L crotonic acid were added to 100 μL of the supernatant and placed at 4°C for 30 min. After centrifuging at 12,000 rpm for 10 min, 100 μL of ethanol was added to100 μL of supernatant then filtered through a 0.22 μm filter membrane for gas chromatography (GC) determination (X). SCFA concentration (M, mmol/L) of sample was calculated as below:


M=X×2×1.2125

The standards of different concentrations were prepared as acetic acid (3.575, 3.660, 2.745, 1.830, and 0.915 mmol/L), propionic acid (4.256, 3.405, 2.554, 1.703, and 0.851 mmol/L), and butyric acid (1.385, 1.108, 0.831, 0.554, and 0.277 mmol/L) with the same concentration of internal standard crotonic acid (1.13125 mmol/L) (19). Then a standard curve was established.

The samples were analyzed using a Varian CP-3800 gas chromatograph (Palo Alto, CA, United States) equipped with an HP-FFAP column (30 m × 0.535 mm × 1 μm) and a flame ionization detector (FID). 1 μL sample was injected at 250°C with a detector temperature of 300°C and nitrogen as the carrier gas at a speed of 35 mL/min and a split ratio of 50:1. The column temperature increased from 100 to 190°C with 20°C/min and then maintained for 1 min (20).

### Statistical analysis

All data were analyzed by SPSS (version 20, SPSS Inc., CO, United States) with the one-way analysis of variance (ANOVA) by LSD test, and expressed as mean ± standard error of mean (SEM). The difference was considered statistically significant at *P* < 0.05.

## Results and discussions

### Comparison of polysaccharide fractions from different species of *Polygonati Rhizoma*

**FIGURE 1:**
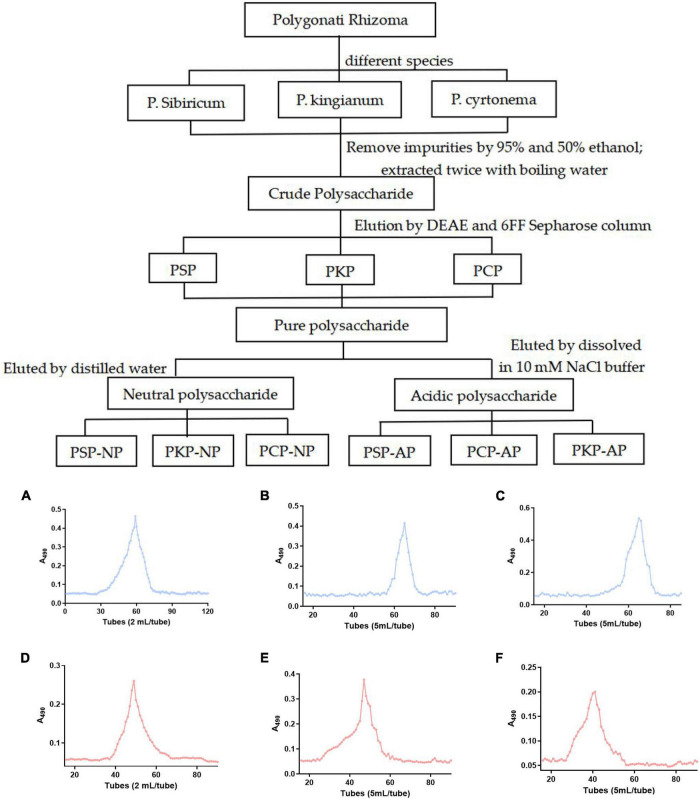
Technical roadmap for extraction and purification of Polygonatum Polysaccharide from different varieties and elution profiles of polysaccharides in gel column. **(A)** PSP-NP, **(B)** PCP-NP, **(C)** PKP-NP, **(D)** PSP- AP, **(E)** PCP-AP, **(F)** PKP-AP.

To obtain the polysaccharide with better biological activity, boiling water extraction was applied to obtain crude polysaccharide from a different resource of *Polygonati Rhizoma*. As shown in Figure 1 and Table 3, the yield of crude polysaccharides varied from 10.3 mg/g to 37.0 mg/g under the same extraction conditions, and the crude polysaccharides from *P. sibiricum* exhibited the highest yield than other two species. The yield of polysaccharide in the present study was slightly higher than the yield of 2.5% thrice refluxed with 95% ethanol, distilled water at 90°C then precipitated with EtOH [1:4 (v/v)], but it was a bit lower than the yield of 10% seen in other research (21, 22). The main reason might be different extraction methods, especially when 50% ethanol was used.

**TABLE 3:** The yield of crude polysaccharides and purified fractions from different species of *Polygonati Rhizoma.*

Crude polysaccharides	Yield (mg/g)	Neutral polysaccharide	Yield (mg/g)	Acidic polysaccharide	Yield (mg/g)
PSP	37.0	PSP-NP	17.2	PSP-AP	7.2
PKP	30.8	PKP-NP	8.1	PKP-AP	2.2
PCP	10.3	PCP-NP	1.6	PCP-AP	14.7

As shown in Table 3, after purification by ion-exchange chromatography and gel filtration, the yield of neutral polysaccharide and acidic polysaccharide fractions became different. As it is shown in Table 3, the highest yield of neutral polysaccharide fraction is PSP-NP, while the highest yield of acidic polysaccharide fraction comes from PCP-AP. The yields of PKP-NP and PKP-AP are very low compared to other two species. Therefore, those two fractions are not subjected to the biological test due to their low yield.

**FIGURE 2:**
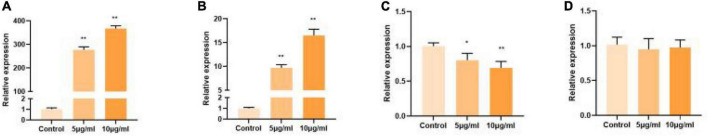
Comparison of polysaccharide fractions from different species of Polygonati Rhizoma. Influence of purified polysaccharides on cell gene expression of SOD1 *in vitro*. **(A)** PSP-NP; **(B)** PSP-AP; **(C)** PCP-AP; **(D)** PCP-NP. *Indicated significant difference compared with a control group, *P* < 0.05; ** indicated significantly.

In previous studies, polysaccharide from Polygonati Rhizoma exhibited potential antioxidant activity *in vitro* (23). Polysaccharides cannot be directly absorbed, but can be degraded by intestinal microorganisms, so the intestine is considered to be the target of polysaccharide activity. Therefore, in the present study, to select the polysaccharide fraction with the highest antioxidant activity for further study, the IPEC-J2 were used as *in vitro* study model. As shown in Figure 2, the polysaccharide fraction PSP-NP exhibited the highest antioxidant activity by promoting the expression of SOD1 *in vitro* compared to other fractions. Thus, the fraction PSP-NP was subjected to structure elucidation and *in vivo* study.

### Structure elucidation of PSP-NP

#### Chemical composition and molecular weight of PSP-NP

The monosaccharide composition of PSP-NP was analyzed by methanolysis and GC analysis. PSP-NP was composed of Gal, Man, and Glc in a ratio of 68.7:24.3:7.0. The results suggested that PSP-NP was mainly composed of Gal and Man, which account for more than 90% of total sugar. From the literatures related the polysaccharide from *P. sibiricum*, they also report the presence of a small amount of Ara except the presence of Man, Glc, and Gal (24–26), while the Ara was not detected in the present study. In another study, the polysaccharide from *P. sibiricum* was composed of Gal and Rha with trace amounts of Man, Glc, and Xyl (27). Additionally, a previous report on the characterization of the other two PSP indicated that one of them only consisted of Gal and another fraction was composed of Gal and Man in a ratio of 88.8:11.2 (28). Collectively, the monosaccharide compositions of PSP-NP are similar with some reports, however, with different ratios and/or compositions.

According to the results of size exclusion chromatography, the standard curve was y−0.0354x + 5.5247,R^2^0.9989, the average molecular weight of PSP-NP was calculated as 43.0 kDa, which was different from those of polysaccharides as 1.4 kDa (29), 8.9 kDa (30), and 76.0 kDa (26) obtained in another research. Five polysaccharides from *P. cyrtonema* were obtained in another study by ethanol precipitation after extraction with hot water and different concentrations of NaOH solutions, and the molecular weights were found to be 2.1, 24.1, 34.3, 38.6, and 42.6 kDa (31), respectively. But the structure of the polysaccharide extracted by water was different from those extracted by NaOH. The molecular weights of polysaccharides obtained from *P. kingianum* were identified to be 8.1, 134.7, 160, and 178.6 kDa (32–34). It suggested that the molecular weights of polysaccharides from different origins of Polygonati Rhizoma varied. The polysaccharide extracted by water with the molecular weight of 43.0 kDa was not found in other studies.

#### Chemical composition and molecular weight of PSP-NP

Linkage analysis showed that 4 methylation-derived glycosyl ion fragments were detected from hydrolyzed fragments of PSP-NP. These are T-Man, 1,4-Gal, 1,4-Glc, and 1,4,6-Gal, being quite different from other studies. Polysaccharide from *P. sibiricum* was found to be composed of 1→6 and 1→2,6-Gal and T-Man (26). T-Man was the common composition in the two studies, but there was also a significant difference in glycosidic linkages of Gal. Research also showed that the main chain of polysaccharides from *P. sibiricum* was composed of 1→4Man and 1→4Glc (25). Besides, T-Gal (35) and 1→3Glc (36) were also detected in other studies. The large differences in the structure of PSP might be due to different extraction conditions or gel column purification.

#### FT-IR and NMR analysis

**FIGURE 3:**
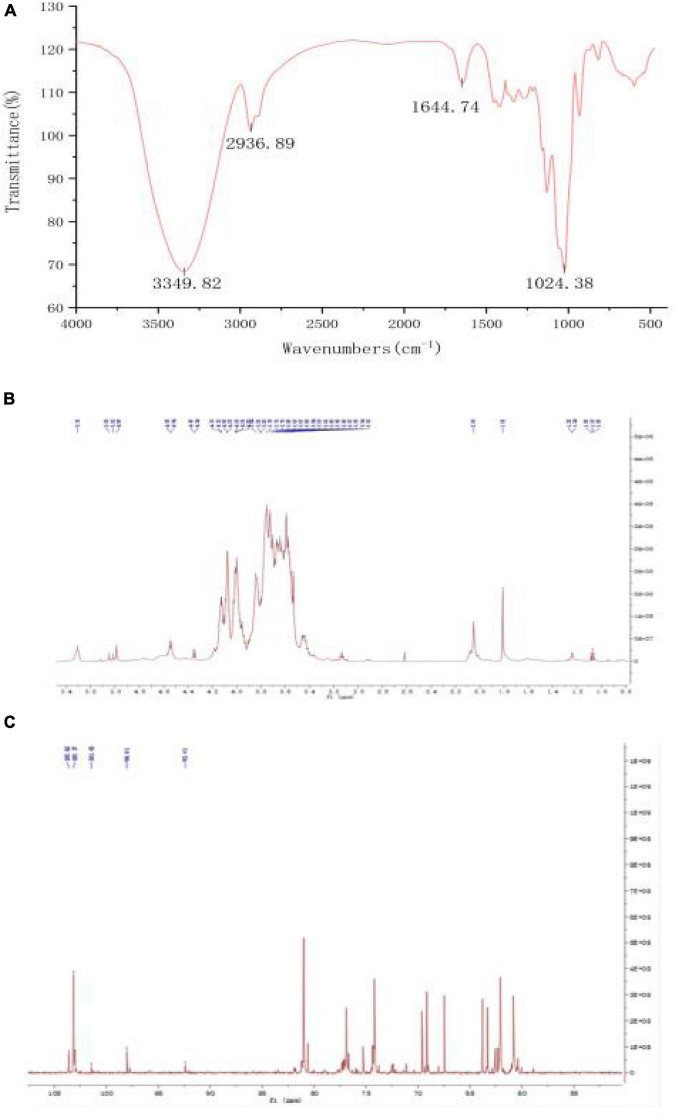
Structure elucidation of PSP-NP. **(A)** FT-IR spectrum. **(B)** 1H NMR spectrum. **(C)** 13C NMR spectrum.

The FT-IR chromatogram of fraction PSP-NP showed characteristic absorptions of polysaccharides (Figure 3A). The absorption at 3349.82 cm^–1^ indicated the presence of O-H stretching (37). The band at 2936.89 cm^–1^ contributed to C-H stretching and bending vibrations of C-H, CH2, and CH3 (38). The stretching peak at 1024.38 cm^–1^ was due to the presence of C-O glycosidic bonds, suggesting the presence of a pyranoid ring (39).

1D NMR spectroscopy was carried out to further study the structure of PSP-NP. The chemical shift values were analyzed by referring to published literature (28, 29, 40). The 1H spectrum (Figure 3B) showed five-strong signals in the chiral carbon region, namely δ 5.35 ppm, δ 5.05 ppm, δ 5.02 ppm, δ 4.99 ppm, and δ 3.55 ppm. It indicated that both α− and β−glycosidic bonds in PSP-NP. δ 5.35 ppm were anomeric protons of the 1,4-α-D-Glc residue, and δ 5.05 ppm was an anomeric proton of the T-α-D-Man residue, while δ 5.02 ppm belonged to T-α-D-Glc. δ 4.99 ppm and δ 3.55 ppm belonged to the anomeric proton with 1,4-β-D-Gal and 1,4,6-β-D-Gal residues, respectively. The rest of the signals belonged to H2∼H6.

In the 13C spectrum (Figure 3C), there were 103.62 ppm, 103.17 ppm, 101.73 ppm, 98.01 ppm, and 92.41 ppm in the chiral carbon region, belonging to 1,4-β-D-Gal, 1,4,6-β-D-Gal, T-α-D-Man, 1,4-α-D-Glc, and T-α-D-Glc, respectively. The rest of the signals belonged to C2∼C6.

The results of the structural analysis showed that PSP-NP was a neutral heteropolysaccharide composed of 1,4-β-D-Gal, 1,4,6-β-D-Gal, T-α-D-Man, 1,4-α-D-Glc, and T-α-D-Glc.

### PSP-NP improves intestinal integrity of aged mice

**FIGURE 4:**
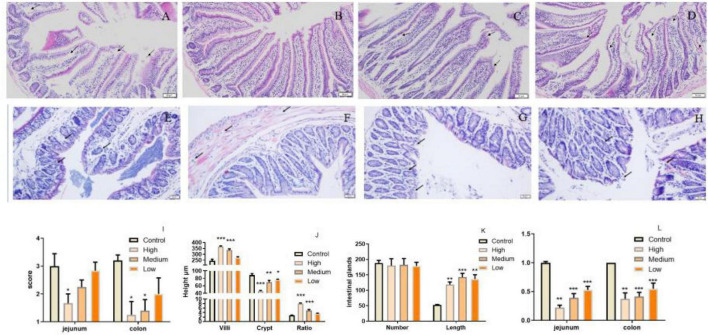
PSP-NP improves intestinal integrity of aged mice. **(A–D)** HE stain of jejunum in the control, high, medium and low dose groups, respectively. Arrows indicated the gaps between villi and epithelial cells. It showed that severe shedding of intestinal villi epithelium and gaps at the top of the villi were improved after drug administration. Bar: 200 μm; **(E–H)** HE stain of colon in the control, high, medium and low dose groups, respectively. Arrows indicated the epithelium and intestinal glands. It showed that epithelium dysplastic and intestinal glands were improved after drug administration. Bar: 200 μm; **(I)** The degree of microscopic injury was evaluated by mucosal and inflammatory conditions from a previously published and wildly used scoring system. The jejunum and colon scores decreased as the concentrations of PSP-NP increased and showed remarkable changes in the high group. **(J)** The length of villi and the depth of crypt in HE stain slice were analyzed by Image-Pro Plus, and the ratios were compared. PSP-NP increased the villi length and suppressed the crypt depth, thus heightening the ratio of villus length/crypt depth in jejunum. **(K)** The number and the length of Colonic glands in HE stain slice were analyzed by Image-ProPlus. PSP-NP increased the length of intestinal glands in colon. There was no significant effect on the number of intestinal glands. **(L)** Gene expression of VIP measured by qRT-PCR in jejunum and colon was significantly reduced in all three doses of PSP-NP. Control indicated control group administrated with saline; and High, Medium and Low indicated high, medium and low dose groups administrated with 200/100/50 mg/kg PSP-NP, respectively. Error bars indicated SEM. *N* = 8. All data were analyzed by SPSS with the one-way analysis of variance (ANOVA) by LSD test, *P < 0.05, **P < 0.01, ***P < 0.001, compared with the control group.

*Polygonati Rhizoma* was reported to be efficacy in promoting immune response and protecting against aging. The intestine is critical for body immunity and sensitivity to aging progress (41, 42). To determine whether neutral polysaccharides from Polygonati Rhizoma play a role in these processes, we supplied PSP-NP to aged male mice (1-year-old) with different doses and evaluated its effects on intestinal immune function. No statistical difference of bodyweight was found after PSP-NP supplement (Data not shown), and the jejunum of small intestine and colon of large intestine were selected to study the benefits of PSP-NP to intestine. We first focus on the morphology integrity of intestine, which is fundamental for intestinal function and immunity (43, 44). Histologically, while observed defects in intestinal integrity like jejunal villi fracture/fall-off and colonic glands deficiency could be found in aged mice, PSP-NP supplement remarkably improves these phenotypes in a dose-dependent manner (Figures 4A–H). These improvements were also revealed by increased “intestinal mucosal injury score” in PSP-NP supplied mice (Figure 4I). In addition to this, we quantified the height of jejunal villi and depth of crypt, finding increased villi height and decreased crypt depth, as well as increased ratio of villus length/crypt depth in PSP-NP supplied mice (Figure 4J), which suggest enhanced functions like secretion, villi epithelial cells regeneration, digestion and absorption of jejunum (45, 46). Meanwhile, PSP-NP enhanced the length of colonic glands (Figure 4K). Congruent with this, in jejunum and colon, we found PSP-NP suppressed the expression of critical neuropeptide—VIP (47) that has the effects on vasodilation and smooth muscle relaxation, also suggesting improvement of intestinal function (Figure 4L).

**FIGURE 5:**
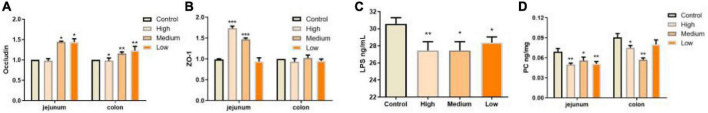
PSP-NP improves intestinal integrity of aged mice. **(A)** Gene expressions of tight junction protein Occludin in jejunum were up-regulated in jejunum but down-regulated in colon. *N* = 8. **(B)** Gene expressions of ZO-1 in jejunum and colon, which were both up-regulated. *N* = 8. **(C)** The LPS contents in serum (ng/mL) were analyzed by ELISA, and it was remarkably decreased after administration of PSP-NP, *N* = 16. **(D)** The content of carbonylated proteins (ng/mg) in jejunum and colon was determined by ELISA, and it was statistically reduced by PSP-NP in all the three groups of PSP-NP. Control indicated the control group administrated with saline, and High, Medium and Low indicated the high, medium and low dose groups administrated with 200/100/50 mg/kg PSP-NP, respectively. Error bars indicated SEM. *N* = 16. All data were analyzed by SPSS with the one-way analysis of variance (ANOVA) by LSD test, *P < 0.05, **P < 0.01, ***P < 0.001, compared with the control group.

Except for morphology integrity, the intestinal barrier integrity exhibited by epithelial cells performs pivotal roles against external factors and is an important part of intestinal immunity (43). Intestinal epithelial cells-expressed tight junction proteins, including occludin, claudins, zonula occludens (ZOs), etc., are crucial for maintaining epithelial barrier integrity (48). In our study, we found that, especially in the jejunum, PSP-NP could promote the expressions of occludin and ZO-1 in aged mice, suggesting improved barrier function (Figures 5A,B). Consistently, in PSP-NP supplied mice, we detected much less concentration of serum LPS, which could be a result of increased intestinal permeability that is affected by aging progress (49; Figure 5C). Oxidative stress and damages are thought to be one of the most important pathological characteristics affecting intestinal permeability in aging progress (3, 50, 51). We observed decreased level of intestinal protein carbonyl, an irreversible and oxidatively modified protein product (52), in jejunum and colon of PSP-NP supplied mice (Figure 5D), indicating PSP-NP functions with benefits to intestine by mitigating aging-induced oxidative stress. Collectively, these data reveal that PSP-NP could improve defects in morphology, barrier integrity and function of intestine of aged mice.

### PSP-NP enhances the intestinal immunity of aged mice

**FIGURE 6:**
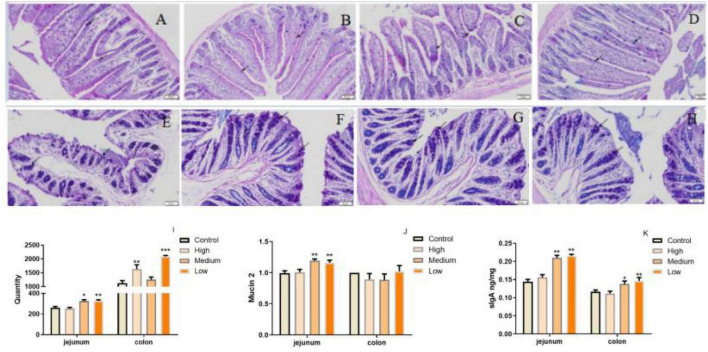
PSP-NP enhances the intestinal immunity of aged mice. **(A–D)** AB-PAS stain of jejunum in the control, high, medium and low dose groups, respectively. Arrows indicated the goblet cells. It showed the goblet cells in jejunum treated with different doses of PSP-NP. Bar: 200 μm. **(E–H)** AB-PAS stain of colon in the control, high, medium and low dose groups, respectively. Arrows indicated the goblet cells. It showed the goblet cells in colon treated with different doses of PSP-NP. Bar: 200 μm. **(I)** The goblet cell count of jejunum and colon in the control, high, medium and low dose groups, respectively, it showed the goblet cells increased as the concentrations of PSP-NP increased and showed remarkable changes in the high group. *N* = 4. **(J)** The Gene expression of Mucin 2 in jejunum and colon was measured by qRT-PCR. The quantification showed that PSP-NP up-regulated gene expression of Mucin 2 in jejunum, there is no significant effect in the colon. *N* = 4. **(K)** The content of sIgA in jejunum and colon was analyzed by ELISA. PSP-NP improved the content of sIgA (μg/mg) in the jejunum and colon in the medium and high dose groups. *N* = 8. Error bars indicated SEM. All data were analyzed by SPSS with the one-way analysis of variance (ANOVA) by LSD test, *P < 0.05, **P < 0.01, ***P < 0.001, compared with a control group.

Considering PSP-NP enhances intestinal integrity and function, it is predicted that PSP-NP may have good benefits to the intestinal immunity of aged mice. To address this issue, AB-PAS staining was first performed to quantify the goblet cells is important for intestinal immunity at mucosal surfaces (53), and the expression of Mucin that is a critical secreted protein from goblet cells that forms a hydrophobic mucus layer on the mucosal surface (54), was then detected. We found that PSP-NP increased the goblet cells’ number in jejunum and colon (Figures 6A–I), and promoted the expression of Mucin in jejunum (Figure 6J). Besides, another secretory immunoglobulin—sIgA was also increased in jejunum and colon of PSP-NP supplied mice (Figure 6K). These results reveal the effects of PSP-NP on improving intestinal mucosal immune response.

**FIGURE 7:**
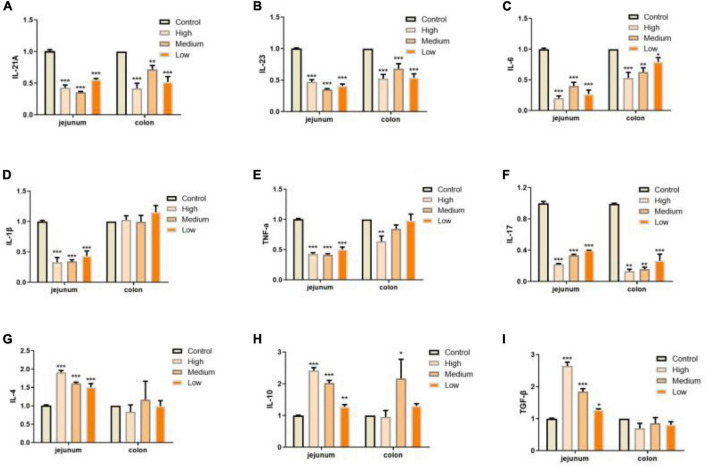
PSP-NP enhances the intestinal immunity of aged mice. **(A–I)** Gene expressions of IL-12A, IL-23, IL-6, IL-1β, TNF-α, IL-17, IL-4, IL-10, and TGF-β measured by qRT-PCR in jejunum and colon, respectively. PSP-NP significantly down-regulated the gene expressions of pro-inflammatory factors including IL-12A, IL-23, IL-6, IL-1β (No significant effect in colon), TNF-α and IL-17, and up-regulated anti-inflammatory factors involving IL-4, IL-10, and TGF-β. Control indicated the control group administrated with saline, and High, Medium and Low indicated high, medium and low dose groups administrated with 200/100/50 mg/kg PSP-NP, respectively. Error bars indicated SEM. *N* = 8. All data were analyzed by SPSS with the one-way analysis of variance (ANOVA) by LSD test, *P < 0.05, **P < 0.01, ***P < 0.001, compared with a control group.

In addition to defects in intestinal integrity, permeability, inflammation is another phenotype in intestine along with aging progress (55). In our study, we evaluated the expressions of several pro-inflammation cytokines (IL-12A, IL-23, IL-6, IL-1β, TNF-α, and IL-17) and anti-inflammation cytokines (IL-4, IL-10, and TGF-β; 55–57) in jejunum and colon of mice from different groups. We found that PSP-NP could significantly suppress the expressions of inflammation factors and promote the expressions of anti-inflammation factors, suggesting the anti-inflammation bioactivity of PSP-NP in intestine (Figures 7A–I). During inflammatory progress, the initial CD4+ T lymphocytes were stimulated by antigen signals, and then differentiated into different T lymphocytes under different conditions, including traditional Th1 and Th2 type effector cells, helper T-cells17 (Th17), and regulatory T-cell (Tregs) (58). The balance of Th1/Th2 cells and Th17/Treg cells are important for intestinal function and immunity. They affect each other, restrict each other, and form a network to maintain a dynamic balance through secreted cytokines (58). IL-12 stimulates the proliferation of activated T cells and promotes the differentiation of Th0 cells to Th1 cells, while Th1 cells secrete IL-6 and further strengthen Th1 cell differentiation (59–61). Th2 cells secrete IL-4 and IL-10, and lymphocytes are mainly differentiated into Th2 cells when IL-4 is highly expressed (60). In this study, IL-12 and IL-6 decreased, while IL-4 and IL-10increased under the treatment of PSP-NP, indicating that the balance of Th1/Th2 trended toward Th2. The presence of Th17 cells can promote inflammation in tissue, while Treg Cells inhibit autoimmunity and inflammation in inflammatory diseases (62, 63). Therefore, the balance between Th17 and Treg cells is crucial. High expression of TGF-β initiates Treg differentiation, which will induce Th17 to highly express both TGF-β and IL-6, and the IL-23 secreted by Th17 maintains the stability and maturity of Th17 in later stages of differentiation (64). In this study, PSP-NP suppressed the expression of interleukins related to Th17, including IL-23, IL-6, IL-1β, TNF-α, and IL-17, while the gene expression of IL-4, IL-10, and the TGF-β expression secreted by Treg cells was increased under PSP-NP treatment. These results suggest that the balance of Th17/Treg cells was also implicated in intestinal inflammatory progress affected by PSP-NP supplement in aged mice.

### PSP-NP improves the compositions of gut microbe and short chain fatty acid

**FIGURE 8:**
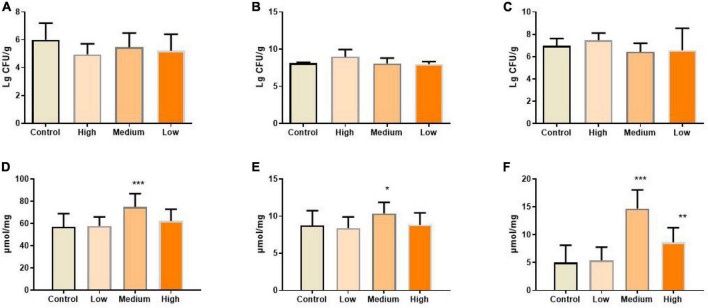
**(A)** The amount of *Escherichia coli* in the content of the intestine was calculated after diluted with 10-fold dilutions of saline from 102 to 107 and cultured in EMB agar medium. It was expressed as. It was reduced by PSP-NP, but showed no significance. **(B)** Bifidobacteria in the content of intestine was cultured in MRS and the amount of Bifidobacteria was calculated and expressed the same as panel **(A)**. It was significantly increased in the high dose group of PSP-NP. **(C)** Lactobacillus in the content of intestine was cultured in BS and the amount of Lactobacillus was calculated and expressed the same as panel **(A)**. The results showed that PSP-NP improved the amount of Lactobacillus. **(D−F)** The contents of acetic acid, propionic acid and butyric acid, respectively, were analyzed by GC. They were all remarkably increased, and the middle dose group showed the best activities. Control indicated the control group administrated with saline, and High, Medium, and Low indicated the high, medium and low dose groups administrated with 200/100/50 mg/kg PSP-NP, respectively. Error bars indicated SEM. *N* = 16. All data were analyzed by SPSS with the one-way analysis of variance (ANOVA) by LSD test, *P < 0.05, **P < 0.01, ***P < 0.001, compared with the control group.

Gut bacteria are crucial for maintaining immune and metabolic homeostasis and protecting against pathogens (65), and multiple polysaccharides were reported to be involved in regulating gut microbe homeostasis (62, 66). Aging progress was reported to affect the intestinal microbial diversity shrinks, reducing the number of beneficial bacteria and increasing facultative anaerobic bacteria (67). For example, higher amounts of Enterobacteriaceae and Coccus were found in aged subjects, while the amounts of Bifidobacterium and Lactobacillus reduced significantly (68, 69). Also, the gut microbiome implicates in age-related inflammation and intestinal permeability defects (55). To determine whether the benefits of PSP-NP to intestinal function and immunity are due to its effects on regulating the composition of gut bacteria, the level of Bifidobacterium, Lactobacillus, and *Escherichia coli* in the gut contents were quantified. Results showed that the population of Bifidobacterium and Lactobacillus increased, while *Escherichia coli* decreased in PSP-NP-supplied mice (Figures 8A–C). Congruent with improvements in gut microbe homeostasis, the levels of three kinds of short-chain fatty acids (SCFAs), which is produced by gut microbes and important immune signal molecules, are increased in gut of aged mice supplied with PSP-NP (Figures 8D–F). This result is consistent with the findings showing polysaccharides from *P. kingianum* also increase the amount of Lactobacillus in gut (32, 70). As Bifidobacterium and Lactobacillus were reported to promote the secretion of sIgA, we think that the increased level of sIgA in PSP-NP supplied mice would be due to enrichment of Bifidobacterium and Lactobacillus populations (71, 72). In addition, Bifidobacterium and Lactobacilli are also implicated in mediating Treg/Th17 balance toward Treg, which affects the balance of cytokines (73). Therefore, improvements in microbe homeostasis defects could be the fundamental mechanism regulating intestinal function and immunity by PSP-NP.

## Conclusion

In this study, one neutral polysaccharide with a high yield was obtained from rhizomes of *P. sibiricum*. PSP-NP was characterized as a neutral heteropolysaccharide composed of 1,4-β-D-Gal,1,4, 6-β-D-Gal, T-α-D-Man, 1, 4-α-D-Glc, and T-α-D-Glc with a molecular weight of 43.0 kDa. Experiments on aged mice *in vivo* showed that PSP-NP could regulate the composition of intestinal microbiome, and promote short chain fatty acids generation to increase intestinal morphology integrity and barrier function, enhance immune function, inhibit inflammation in the intestine of aged mice, which will be valuable for its further applications on the treatments of aging-related intestinal diseases.

## Data availability statement

The original contributions presented in this study are included in the article/supplementary material, further inquiries can be directed to the corresponding author.

## Ethics statement

This animal study was reviewed and approved by the Animal Care and Use Committee of Sichuan Agricultural University.

## Author contributions

MT-T carried out the data curation. XF carried out the formal analysis. L-XL carried out the funding acquisition. BF and WL investigated the data. CH, XZ, and L-ZY performed the methodology. Y-FZ carried out the project administration. BP carried out the resources. X-XL and H-QT carried out the software. Z-QY supervised the data. BF and XS validated the data. L-XL and XF wrote the original draft of the manuscript. M-TT and CH wrote, reviewed, and edited the manuscript. All authors read and agreed to the published version of the manuscript.
